# Changes in Plasma Acylcarnitine and Lysophosphatidylcholine Levels Following a High-Fructose Diet: A Targeted Metabolomics Study in Healthy Women

**DOI:** 10.3390/nu10091254

**Published:** 2018-09-06

**Authors:** Anita Gonzalez-Granda, Antje Damms-Machado, Maryam Basrai, Stephan C. Bischoff

**Affiliations:** Institute of Clinical Nutrition, University of Hohenheim, Fruwirthstr 12, 70599 Stuttgart, Germany; anita.gonzalez_granda@uni-hohenheim.de (A.G.-G.); a.damms-machado@uni-hohenheim.de (A.D.-M.); m.basrai@uni-hohenheim.de (M.B.)

**Keywords:** fructose, targeted metabolomics, metabolic syndrome, liver disease

## Abstract

Background: The consumption of high amounts of fructose is associated with metabolic diseases. However, the underlying mechanisms are largely unknown. Objective: To determine the effects of high fructose intake on plasma metabolomics. Study design: We enrolled 12 healthy volunteers (six lean and six obese women, age 24–35 years) in a crossover intervention study. All participants carried out three diets: (1) low fructose (<10 g/day); (2) high fructose (100 g/day) from natural food sources (fruit); and (3) high fructose (100 g/day) from high fructose syrup (HFS). Outcome measures: The primary outcome was changes in plasma metabolites measured by targeted metabolomics. Results: High compared to low fructose diets caused a marked metabolite class separation, especially because of changes in acylcarnitine and lysophosphatidylcholine levels. Both high fructose diets resulted in a decrease in mean acylcarnitine levels in all subjects, and an increase in mean lysophosphatidylcholine and diacyl-phosphatidylcholine levels in obese individuals. Medium chain acylcarnitines were negatively correlated with serum levels of liver enzymes and with the fatty liver index. Discussion: The metabolic shifts induced by high fructose consumption suggest an inhibition of mitochondrial β-oxidation and an increase in lipid peroxidation. The effects tended to be more pronounced following the HFS than the fruit diet.

## 1. Introduction

Recent data from the Organisation for Economic Co-operation and Development (OECD) and Food and Agriculture Organisation of the United Nations (FAO) reported an increased consumption of high fructose corn syrup (HFCS) in the European Union and predicted a dramatic rise of 225% by the year 2025 [1]. In the United States, the consumption of HFCS, which is often used to sweeten soft drinks, has increased enormously [2]. At the same time, the prevalence of obesity has grown as well, suggesting an association between high fructose use and an increase in body weight in the population [2,3]. HFCS, consisting of a minimum of 55% fructose, the remaining part being glucose, is made from corn starch. It is often called corn syrup or fructose-glucose syrup. Today, fructose delivered from HFCS accounts for about half of the sugar intake in the US. The daily intake of energy derived from fructose rose to an average of 324 kcal per person [3], which corresponds to approximately 12% of the total energy intake. In Europe, on the other hand, predominantly beets-derived sucrose, also known as glucose-fructose or fructose-glucose syrup, is added, which contains the same monosaccharides, however usually in a (40–60%):(40–60%) ratio.

Fructose occurs as an intermediate of the glucose metabolism, but is not an essential nutrient for human metabolism. The hepatic metabolism of fructose bypasses the rate-limiting step of glycolysis and may cause increased glycogen formation, de novo lipogenesis, and lactic acidosis [4]. Animal models have shown that chronic fructose intake induces dyslipidaemia and insulin resistance [5], but only moderate obesity [6,7]. In humans high fructose intake was reported to have significant effects of metabolic syndrome related diseases, including an increased risk of dyslipidemia and insulin resistance [8,9]. Anyhow, results from a meta-analysis investigating the effect of fructose uptake on insulin sensitivity concluded that fructose consumption induces hepatic insulin resistance in normal weight, nondiabetic subjects, but short- and medium-term fructose uptake does not promote insulin resistance [10]. 

In recent years, the endocrine and metabolic effects of free fructose have been compared with those of glucose, sucrose, and also HFCS [11,12,13,14]. Consumption of a high fructose diet, in contrast to a high glucose diet, stimulates the pathological characteristics associated with the metabolic syndrome namely increased visceral fat, dyslipidemia, and insulin resistance [11]. Schaefer et al. [12] reported that a fructose consumption of 20–25% of the energy intake resulted in elevated fasting triglyceride levels as well as low-density lipoprotein (LDL) levels, whereas the same amount of glucose consumption did not induce these effects. It has been shown that consuming fructose-sweetened beverages at mealtimes compared to glucose-sweetened beverages leads to lower insulin secretion, lower daily profiles of leptin, and increased postprandial triglyceride concentrations in obese patients [14]. The latter were mainly observed in obese subjects with insulin resistance, suggesting that fructose intake may aggravate an unfavorable metabolic profile found in obese individuals [14]. Contrary results were reported by Chiavaroli et al., who concluded from a meta-analysis that fructose does not have adverse effects on blood lipids [15].

The underlying mechanisms by which a high fructose diet causes metabolic alterations, are not fully understood. We hypothesized that a high fructose diet causes changes in the metabolome that might be related to the metabolic changes. To test this hypothesis, we conducted the first human pilot trial, in which we examined the effects of high fructose diets on the plasma metabolome in young and healthy adults. About 3/4 of the fructose intake came from sugar-supplemented foods and drinks, the remaining 1/4 from natural fruits and vegetables [16]. Therefore, we were also interested to see if the source of fructose is relevant for the metabolic changes and evaluated the volunteers with fructose administered either by high fructose syrup (HFS) or fructose derived from fruits and vegetables. 

## 2. Methods 

### 2.1. Study Design

**Figure 1:**
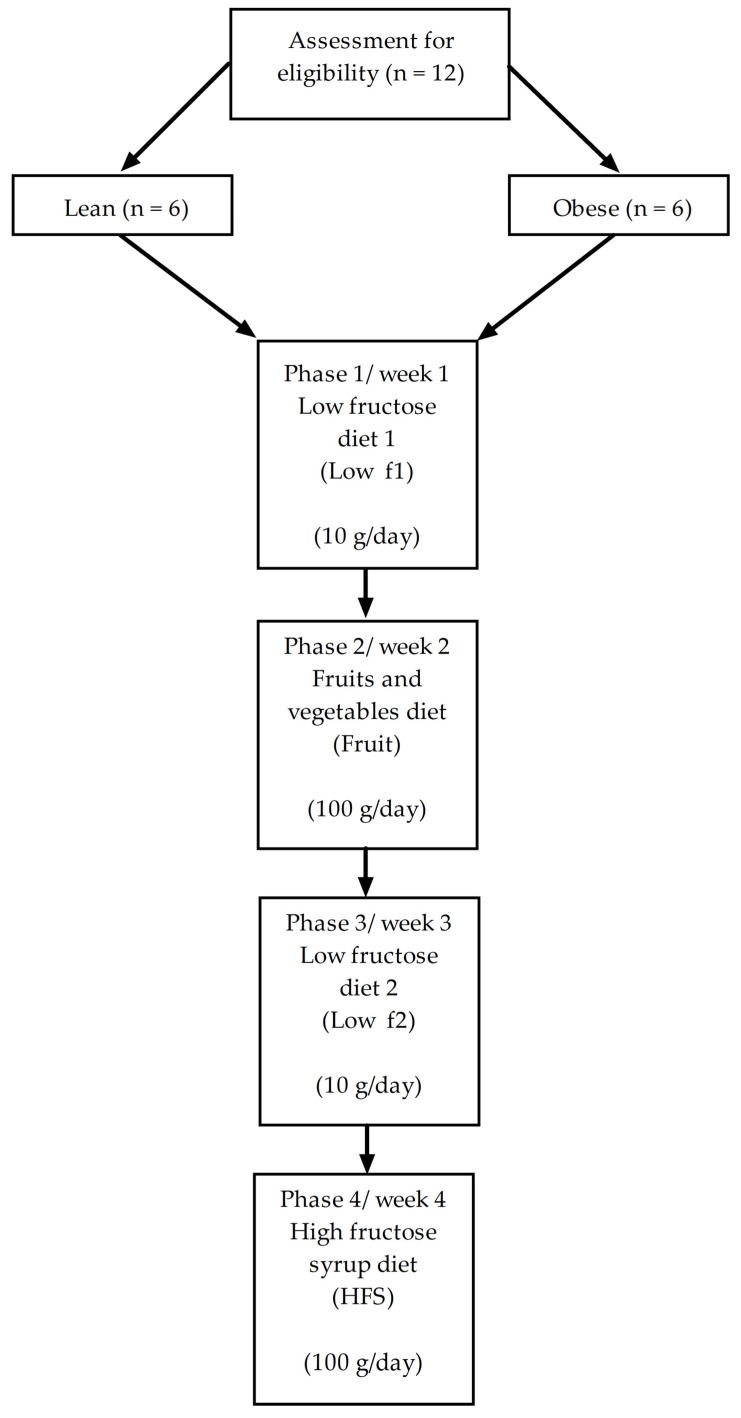
Study design.

A crossover intervention study, in which subjects received a sequence of three diets, was performed. All individuals received first a low fructose diet (1st study phase, labeled “low f1”, serving as a control diet), secondly a high fructose diet rich in fruits and vegetables (2nd study phase, labeled “fruit”), third again a low fructose diet (3rd study phase, labeled “low f2”, serving as a wash-out phase) and forth a high fructose diet rich in HFS (4th study phase, labeled “HFS”, Figure 1). Each diet phase was performed for one week, the whole intervention time was four weeks. 

### 2.2. Selection of Subjects

Twelve healthy, lean and obese volunteers aged between 20 and 40 years who gave a written informed consent were eligible to participate in the study. To reduce interindividual variabilities in fructose responses within the study group, we restricted age to young adults and sex to females. To check for eligibility, a medical history, physical examination, and a blood sample collection (10 mL) was performed. Furthermore, a hydrogen breath test was conducted to detect potential fructose malabsorption [17]. Subjects were recruited through the employees email distribution of the University of Hohenheim over a time period of six weeks. The study was conducted from 2009 to 2010 at the metabolic unit of the University of Hohenheim, in Stuttgart, Germany. The study was approved by the responsible ethics committee (Landesärztekammer Baden-Württemberg, Stuttgart, Germany, no. 2009-079-f) and carried out in accordance with the Helsinki Declaration (revised version, 1989). The study was registered at ClinicalTrials.gov (ID: NCT03444233).

Exclusion criteria were smoking, fructose malabsorption, pregnancy, chronic gastrointestinal diseases (e.g., inflammatory bowel disease, irritable bowel syndrome, celiac disease etc.), gastrointestinal surgery in the past (except appendectomy), chronic anemia, chronic hepatic or renal disease, diabetes mellitus, consuming illnesses such as cancer or HIV, or intake of drugs affecting lipid or glucose metabolism such as statins, weight-reducing or anti-diabetic drugs, antihypertensive drugs, steroids, or antidepressants.

According to their body mass index (BMI), study participants were assigned either to the lean (20 < BMI < 25 kg/m^2^, *n* = 6) or to the obese (35 < BMI < 50 kg/m^2^, *n* = 6) group. Compared to the lean subjects, the obese had a slightly higher age and clearly higher BMI, waist circumference (WC), blood pressure (BP), LDL level, glutamate-pyruvate-transaminase (GPT), also known as alanine aminotransferase (ALT) level, and fatty liver index (FLI) (Table 1).

### 2.3. Dietetic Intervention

At the baseline of the study, all subjects received nutritional advice via group meetings in which they were informed about the fructose content in food. In addition to the group meeting, an individual nutritional counseling based on a diet plan was conducted. Energy needs were determined individually using the Harris-Benedict equation 55 + (9.56 ∗ weight(kg) + (1.85 ∗ height(cm) − 4.68 ∗ age(years)). All three diets were adjusted to isocaloric and isonitrogenous diets with an energy intake of 30% by fat and 15% by protein. The carbohydrate fraction varied by 20% in fructose and 35% in other carbohydrates during the high fructose phases, and 2% in fructose and 53% in other carbohydrates during the low fructose phases (Table 2). 

The subjects received three different diets while they went through four diet phases (Figure 1). During the 1st phase (diet 1; low f1), subjects were allowed to consume a maximum of 10 g of fructose per day, by avoiding added sugar and fruits or vegetables containing more than 1 g fructose per 100 g food, sweets like chocolate, ice-cream, ketchup, juices, soft drinks, and high processed foods. During the 2nd phase (diet 2; fruit), 20% of the energy intake came from fruits and vegetables, corresponding to a fructose intake of about 100 g/day. Dried fruits and natural fruit juices were allowed in moderate amounts. Sugar, sweets, and high processed foods had to be avoided. The diet during the 3rd phase (diet 1; low f2) was again a low fructose phase equal to low f1 described previously. The 4th phase (diet 3, HFS) was another high fructose phase achieved by sweetened food with fructose-glucose syrup (C-TruSweet 01750, Cargill Deutschland GmbH, Krefeld, Germany). Other sources of fructose than the HFS source such as fruits and vegetables as well as sweetened foods were avoided in the 4th phase. In case the participant did not meet the fructose uptake goals of the study diet in the fruit phase, the fructose load during the HFS phase was adjusted to that of the fruit phase to allow better comparison of the two high fructose phases. For assessment of the actual nutritional intake, patients had to fill in a dietary record every day during the study phase. The dietary records were analyzed using the EBISPro software, version 8.0 (Dr. Erhardt, University of Hohenheim, Stuttgart, Germany).

During the low fructose diets, the obese subjects had a higher protein and all subjects a higher fat intake compared to the defined study diet. This can be explained by the reduced carbohydrate intake resulting in more “hunger” for other nutrients. Thus, subjects compensated the low sugar intake by protein and fat rich foods. During the fruit phase the complex carbohydrate uptake was lower compared to the defined target. It can be assumed that subjects preferred to consume fruits rather than vegetables with high fructose, such as grapes, to reach the aimed 100 g fructose per day. The energy intake during the HFS phase was higher than suggested by the defined study diet, possibly because subjects consumed syrup in addition to the proposed diet without reducing other sugar sources. Furthermore, lean subjects showed a reduced intake of complex carbohydrates presumably because of a reduced intake of fructose-rich fruits and vegetables. Not surprisingly but very noticeable was the fiber content, which was significantly higher in the fruit compared to the low fructose and HFS phase.

### 2.4. Outcomes 

The primary outcome was changes in plasma metabolites measured by targeted metabolomics. Blood samples were collected at baseline and weekly after each diet period. The blood sample collection took place between 7:30 and 9:00 a.m. after an overnight fast of 12 h by arm venipuncture. Ethylenediaminetetraacetic acid (EDTA)-plasma was obtained by centrifugation and stored at −80 °C until analysis. Targeted metabolomic analysis and data processing was performed by the working group of Prof. Dr. Adelbert Roscher, University of Munich Medical Centre, Dr. von Hauner Children’s Hospital, Div. Metabolic and Nutritional Medicine. Blood plasma was sent on dry ice to the research laboratory of the Ludwig-Maximilians-University of Munich. In brief, AbsoluteIDQ^®^ p180 Kit (Biocrates Life Sciences AG, Innsbruck, Austria) was used for the metabolic analysis following the manufacturer’s instructions. The metabolite screening was conducted using a tandem mass spectrometry (4000 QTrap^TM^; AB Sciex, Darmstadt, Germany). Flow injection assay (FIA)-MS/MS was used to determine acylcarnitines (*n* = 40), hexoses (*n* = 1) as well as phosphatidylcholines (PC, *n* = 76), lysophosphatidylcholines (lysoPC, *n* = 14) and sphingomyelins (SM, *n* = 15). A liquid chromatography (LC)-MS/MS was applied for the analysis of amino acids (*n* = 21) and biogenic amines (*n* = 16). Quantitative data were obtained using MetIDQ^TM^ Software.

Secondary outcomes were selected laboratory parameters relating to metabolic alterations such as insulin resistance and fatty liver disease measured in a certified medical laboratory (Laborärzte Sindelfingen, Germany) using standard photometry techniques. We determined fasting levels of glucose, glutamic oxaloacetic transaminase (GOT), which is also known as aspartate aminotransferase (AST), GPT, gamma-glutamyl-transpeptidase (GGT), alkaline phosphatase (AP), high-density lipoprotein (HDL), LDL, triglycerides, blood sedimentation rate (BSR), creatinine, urea, and uric acid. Moreover, during the initial medical examination at baseline and weekly after each diet period, anthropometric data (body weight, BMI, WC) and blood pressure were recorded. To assess signs of liver steatosis, sonography was performed using the LOGIQ P6 device (GE Healthcare, Solingen, Germany) by a trained physician as described [18]. Hepatorenal index (HRI) was assessed according to Webb et al. [19] and fatty liver index (FLI) was determined according to Bedogni et al. [20]. 

### 2.5. Statistical Analysis

A sample size calculation was performed using g Power 3.1.9.2 (GPower Software Inc., University of Kiel, Kiel, Germany). On the basis of supervised multivariate classification analysis derived from the Simca software for metabolite analysis (see below), with an α = 0.05 and an effect size of f^2^ = 1.45 resulting from data goodness of fit (R2) and mean square residuals from cross validated (CV) analysis of variance (ANOVA), twelve subjects are needed for reaching a power of 90%. Since the subject number is low and the sample size calculation is poorly established for studies with multiple metabolome data, we declared our study as a pilot trial. 

Statistical analysis was performed using RStudio version 1.1.423 (RStudio, Inc., Boston, MA, USA), R software 3.4.3 (R Core Team, Vienna, Austria) and its packages Hotelling, reshape 2, bindr, ggplot, and heatmap3. Further statistical programs used in the analysis were GraphPad Prism version 5.0 (Graph Pad Software, Inc., La Jolla, CA, USA) and Simca-P+ Version 13.0 (Umetrics, Umeå, Schweden). To test for statistical significant difference between group means in clinical and nutritional parameters, unpaired Student’s *t* test was used for normally distributed data and Mann–Whitney *U* test was carried out for non-normally distributed data. For testing differences within diet phases, paired Student’s *t*-test with Bonferroni correction for multiple testing was used. 

Metabolites, which had a detection frequency of less than 50%, were excluded from the dataset before analysis. Metabolomic data were log transformed to ensure a normally distributed data set. Data were pooled to test between or within low (low f1 + low f2) and high fructose (fruit + HFS) diets. Supervised multivariate classification was performed after UV scaling using orthogonal partial least squared-discriminant analysis (OPLS-DA) [21]. Goodness of fit (R2) and goodness of prediction (Q2) were stated. R2X(cum), R2Y(cum), and Q2(cum) were calculated. R2X(cum) indicates the cumulative (predictive and orthogonal) variation in X (metabolites) which is explained by the model, R2Y(cum) the cumulative variation of Y (samples) explained by the model, and Q2(cum) the cross validated predictive ability of the model. R2X(cum), R2Y(cum), and Q2(cum) describe the quality of the model. In general, high R2 and Q2 are desired while the difference between R2 and Q2 should be as small as possible. A Q2 value above 0.5 can be seen as a good model and is typical in metabolomic research. Cross-validated (CV) ANOVA was performed to test for significant predictive performance of the model. The OPLS-DA scatter plot was used to visualize the class separation. The loading plot as well as Variable Importance for the Projection (VIP) was performed to estimate the contribution of each metabolite and metabolite classes to the class separation (low vs. high fructose phases). Metabolites with a VIP value larger than 1 were considered as metabolites with a high contribution to class separation. ANOVA was performed to test for treatment effects. Paired Student’s *t*-test was performed to test for significant within-person differences between phases in plasma metabolite abundance. To correct for multiple comparisons, the Benjamini-Hochberg (FDR, false discovery rate) method was used. For testing differences in metabolite concentration between groups (obese and lean), an unpaired Student’s *t*-test was carried out. For comparing significant differences between phases in concentrations of metabolites and metabolite classes, data were mean centered and scaled by standard deviation (unit variance (UV) scaling) before analyzing using paired Student’s *t*-test corrected for multiple tests by FDR correction. To analyze differences between mean metabolite classes, FDR correction was performed by ranking 15 medium and long chain acylcarnitine, 15 lysoPCs, and 23 diacyl-phosphatidylcholines (PCaa). To analyze differences in metabolite concentrations, FDR correction was carried out by ranking 183 metabolite concentrations. Correlation heatmap using Pearson correlation was performed to visualize correlations between metabolites and clinical parameters. A dendrogram was performed to show the hierarchical clustering of metabolites and clinical parameters. A *p*-value of <0.05 was seen as statistically significant and a *p*-value between 0.05 and 0.1 was considered as a trend. Results were documented as mean ± SD if not indicated otherwise.

## 3. Results 

Fructose intolerance is common in the general population. Therefore, all subjects were examined for fructose intolerance using the H2-breath test before study inclusion. No one responded by increased H2 exhalation or symptoms. Moreover, no one reported any unintended effects or harms after following the intervention phases of high consumption of fruits or high fructose syrup. 

### 3.1. Fructose Affects the Metabolic Profile in Plasma

**Figure 2:**
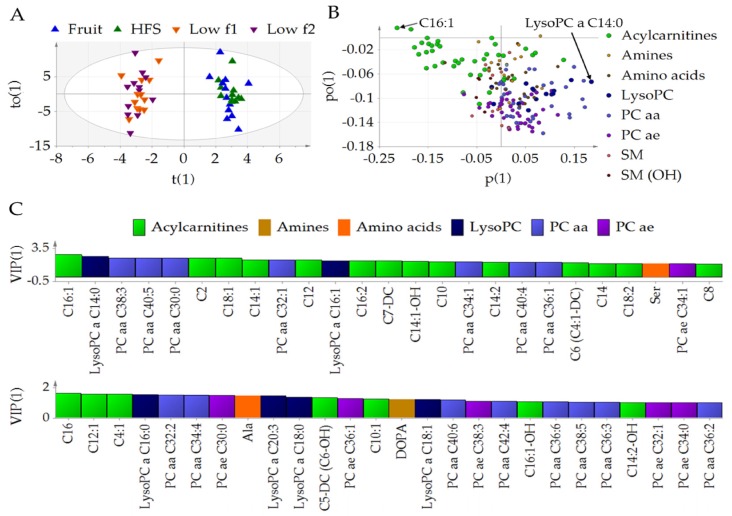
Orthogonal partial least squared-discriminant (OPLS-DA) analysis. Class separation is shown by (**A**) scatter plot, (**B**) loading plot, (**C**) Variable of Importance for Prediction (VIP). t(1), score vector of predictive component 1; to(1), score vector of orthogonal component 1; po(1), loading vector of orthogonal component 1; p(1), loading vector of component 1. Abbreviations: HFS, high fructose syrup; low f, low fructose; lysoPC, lysophosphatidylcholines; PCae, acyl-phosphatidylcholine; PCaa, diacyl-phosphatidylcholine; SM, sphingomyelins.

Acylcarnitine C16:1 (Hexadecenoyl-l-carnitine) (adj. *p* = 0.029) as well as lysoPC a C14:0 (adj. *p* = 0.031) were influenced by the different diets. When pooling the data from the low fructose diets (low f1 + low f2) and those from the high fructose diets (fruit + HFS), the OPLS-DA scatterplot (Figure 2A) shows a clear class separation with low fructose diet data clustering at the left and high fructose diet data at the right corner. The corresponding loading plot (Figure 2B) suggests that acylcarnitines were mainly responsible for the clustering of the low fructose diet and thus were altered in the low fructose phases. LysoPCs contributed mainly to the clustering of the high fructose diet (Figure 2B). Furthermore, acylcarnitine C16:1 seems to be mostly responsible for the clustering of the low fructose diet while lysoPC a C14:0 for the high fructose diet samples. The VIP analysis shows again that acylcarnitine C16:1 contributed mainly to the class separation, followed by lysoPC a C14:0, PCaa C38:3, and PCaa C40:5 (Figure 2C). Further relevant metabolites come from the metabolite classes acylcarnitine, lysoPC, and PCaa. The model was statistically significant with *p* = 0.010 (CV ANOVA), R2X(cum) of 0.597, R2Y(cum) of 0.968, and Q2(cum) of 0.532. R2X (1) of 0.0477, indicating that 4.77% of the variation in X of the first component was related to the separation of low and high fructose diet. The within group variation was explained by 23.1% of the orthogonal component 1 (R2X(o1)). Significant differences of metabolite concentrations between low and high fructose diets are shown in Table S1.

### 3.2. Diet-Dependent Changes of Acylcarnitine, Lysophophadidylcholines, and Diacyl-Phophadidylcholines

**Figure 3:**
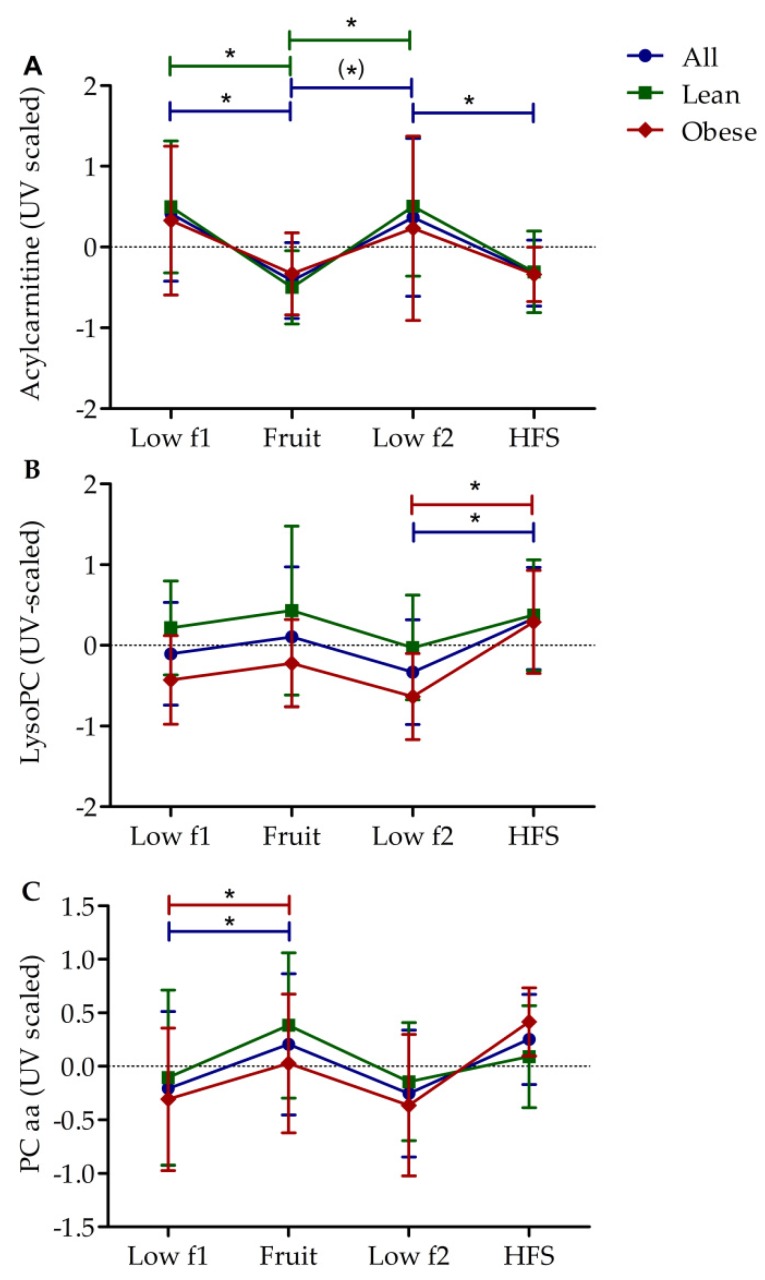
Changes of metabolite levels within study phases. Mean (± SD) of (**A**) medium and long chain acylcarnitine levels, (**B**) lysoPC levels, (**C**) PCaa levels after unit variance (UV) scaling of all (*n* = 12), lean (*n* = 6) and obese (*n* = 6) subjects are shown for four study phases (explained in Figure 1). Significant differences between phases are indicated as * adj. *p* < 0.05. Trends are indicated as (*) adj. *p* < 0.1. Abbreviations: HFC, high fructose syrup; low f, low fructose; lysoPC, lysophosphatidylcholines; PCaa, diacyl-phosphatidylcholine.

Because OPLS-DA analyses showed a high contribution of medium- and long-chain acylcarnitines, as well as lysoPC and PCaa to the class separation of low and high fructose diets (Figure 2B), the mean levels (after UV scaling) of these metabolites were further analyzed for lean, obese, and all subjects in different study phases (Figure 3). Mean acylcarnitine levels (Figure 3A) showed the same pattern in lean, obese, and all subjects. When changing from phases of low to high fructose diet, the acylcarnitine level generally decreased, and when changing from phases of high to low fructose, it increased. This was statistically significant for all diet changes when data from all subjects were pooled and for most diet changes when only lean subjects were analyzed. However, when analyzing only obese individuals, the changes of acylcarnitine levels were not significant (Figure 3A). 

With regard to the mean lysoPC levels (Figure 3B), the diet-induced changes tended to be inverse. High fructose diets caused higher levels of lysoPC than low fructose diets, however, the changes were only significant for HFS in the obese but not in the lean group. 

Mean PCaa levels (Figure 3C) showed a similar trend as described for lysoPCs with the difference that a significant increase in levels was found only in obese subjects on the fruit diet but not on HFS diet.

### 3.3. Metabolic Profile of Single Metabolites

**Figure 4:**
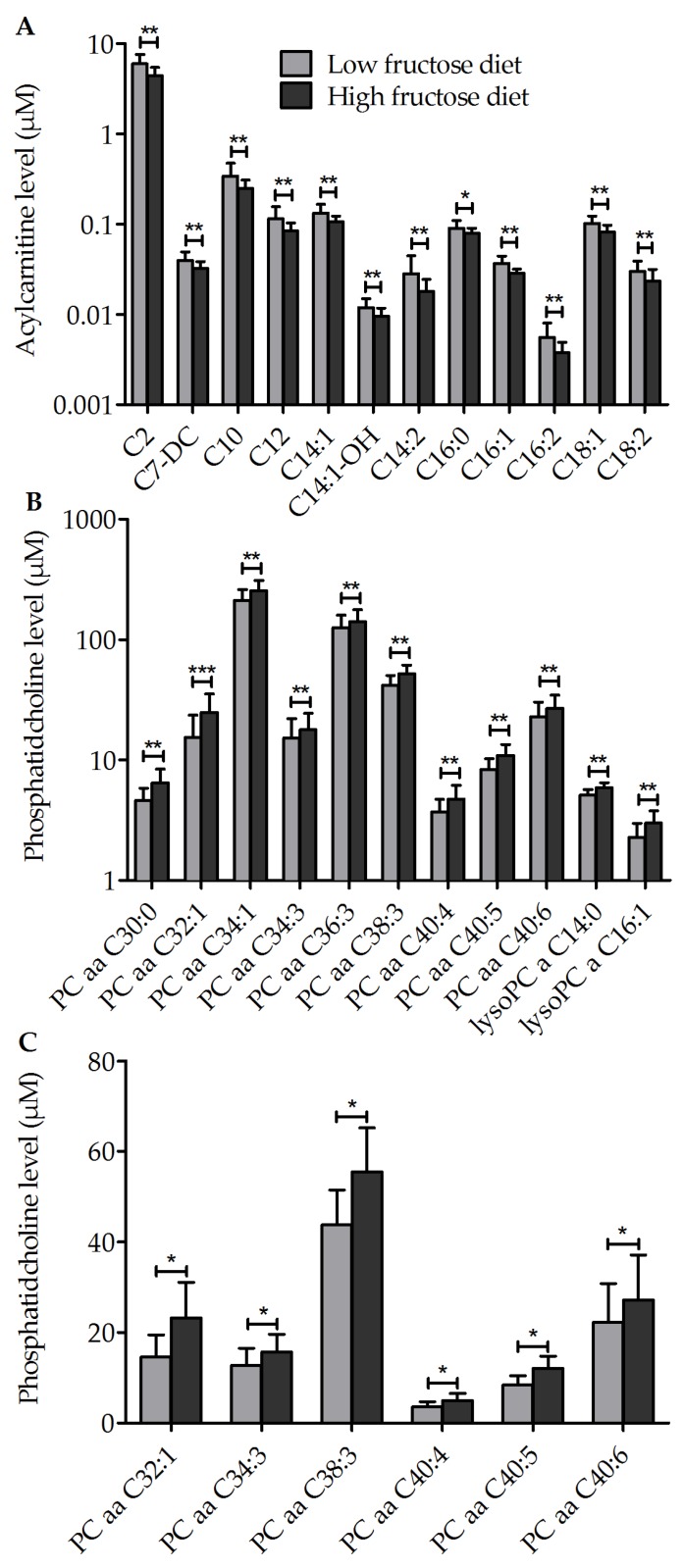
Significant differences in metabolites between the low fructose diet (1 + 2) and high fructose diet (fruit + HFS). (**A**) + (**B**) all (*n* = 12), (**C**) obese (*n* = 6) subjects, data are shown as mean (± SD). Significant differences between low fructose and high fructose phases are indicated as * adj. *p* < 0.05 and ** adj. *p* < 0.01. Abbreviations: lysoPC, lysophosphatidylcholines; PCaa, diacyl-phosphatidylcholine.

Metabolites from four metabolite classes showed significant differences between the low fructose and high fructose diet groups, including acylcarnitines and three types of glycerophospholipids (Figure 4A,B, Table S1). Confirming previous results shown in Figure 2B, the metabolites acylcarnitine C16:1 and lysoPC a C14:0 differed, among others, significantly between the low fructose and high fructose diet groups. Additionally, as described before, acylcarnitine C16:1 and lysoPC a C14:0 showed high class separation (Figure 2B,C). When analyzing obese and lean subjects separately, six particular PCaa metabolites differed between the low fructose and high fructose diet groups in obese (Figure 4C), but not in lean individuals. 

**Figure 5:**
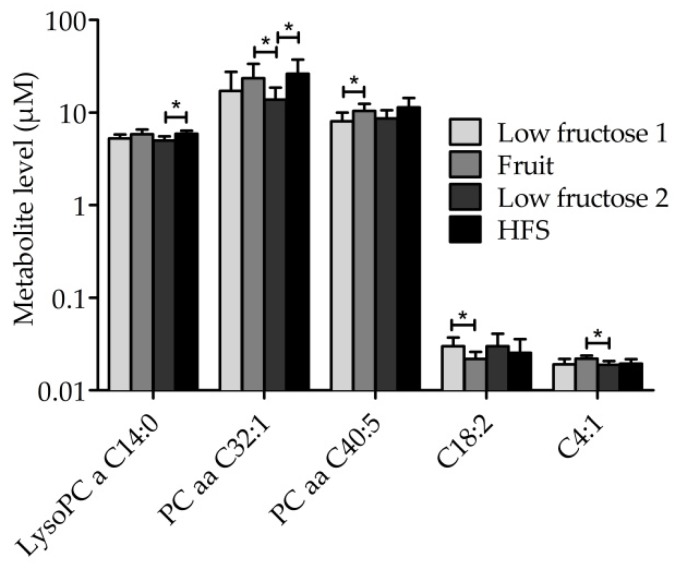
Mean (± SD) of metabolite levels within study phases. Mean (± SD) of lysoPC a C14:0, PCaa C32:1, PCaa C40:5, acylcarnitine C18:2, and acylcarnitine C4:1 of all subjects (*n* = 12) are shown for four study phases (explained in Figure 1). Significant differences between phases are indicated as * adj. *p* < 0.05. Abbreviations: HFS, high fructose syrup; lysoPC, lysophosphatidylcholines; PCaa, diacyl-phosphatidylcholine.

After analyzing differences in metabolite levels in all subjects within the four study phases, we found that there were significant variations in the levels of the phospholipids lysoPC a C14:0, PCaa C32:1, and PCaa C40:5, as well as in the levels of the acylcarnitines C18:2 and C4:1 (Figure 5).

### 3.4. Other Outcomes 

No statistically significant differences were found with regard to laboratory parameters including GOT, GPT, GGT, HDL, LDL, triglycerides, blood sedimentation rate (BSR), creatinine, urea, uric acid, between the dietetic phases examined in the study. Also clinical parameters (body weight, WC, BMI, blood pressure) and markers of liver steatosis (HRI, FLI) were not changed by the diets. 

### 3.5. Heatmap Correlations between Acylcarnitine and Phophatidylcholine Levels, Respectively, and Clinical Parameters

**Figure 6:**
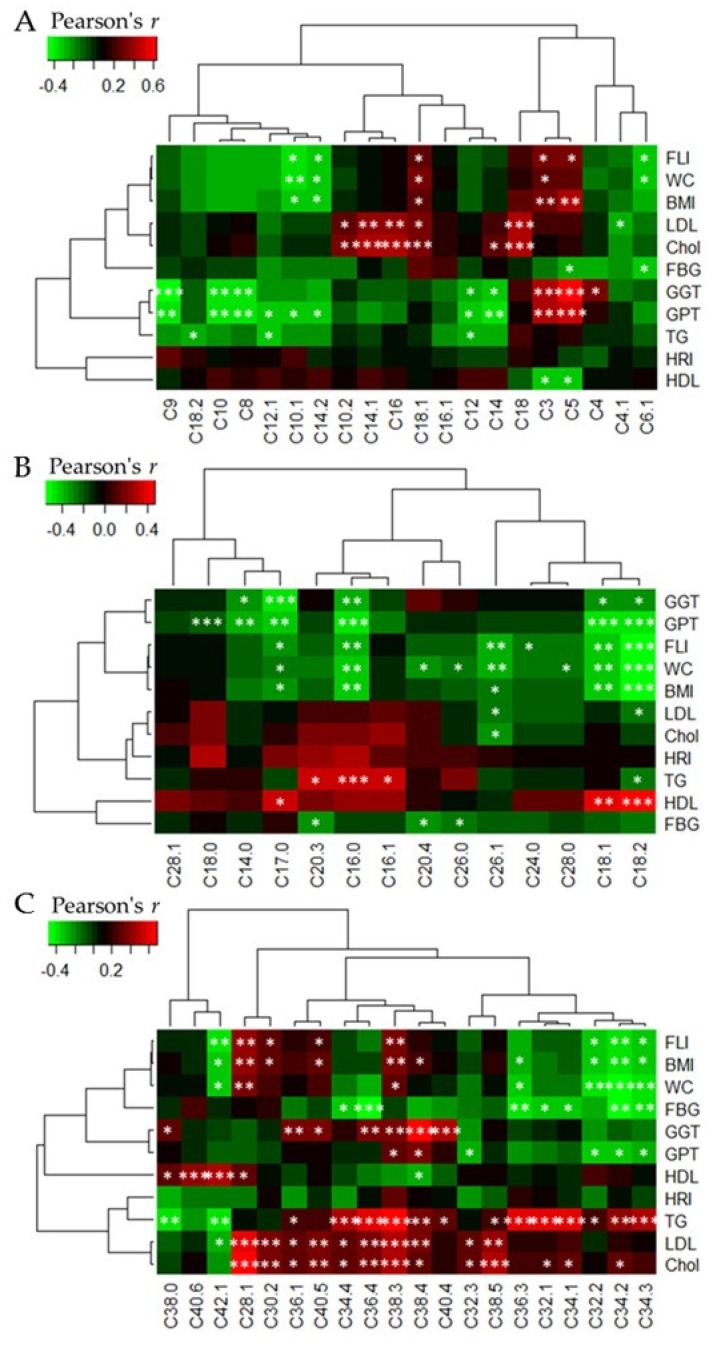
Correlation heatmap (Pearson correlation) of metabolites and clinical parameters. (**A**) Acylcarnitines, (**B**) lysophosphatidylcholines, (**C**) diacyl-phosphatidylcholine. Green colored fields show negative correlation coefficients (*r*) and red colored field positive ones. The dendrograms show the hierarchical clustering of metabolite classes and clinical parameters. Abbreviations: BMI, body mass index; Chol, total blood cholesterol; FBG, fasting blood glucose; FLI, fatty liver index; GGT, gamma-glutamyl-transpeptidase; GPT, glutamate-pyruvate-transaminase; HDL, high density lipoprotein; HRI, hepatorenal index; LDL, low density lipoprotein; TG, triglycerides; WC, waist circumference.

Pearson correlation analysis was performed to test significant correlations between acylcarnitines (Figure 6A), lysoPCs (Figure 6B), and PCaas (Figure 6C) on the one hand and a number of different clinical parameters such as anthropometric parameters and blood parameters on the other. A negative correlation could be found between acylcarnitine C18:2, C12:1, as well as C12:0, and serum triglycerides. Medium- and long-chain acylcarnitines were negatively correlated with the liver enzymes GPT and with GGT whereby short-chain acylcarntines correlated positively with GPT and GGT. Furthermore, positive correlations were found between short-chain acylcarnitines C3 as well as C5 and FLI. Furthermore, acylcarnitines C3 as well as C5 correlated positively with BMI and acylcarnitine C3 correlated positively with WC (Figure 6A). Five lysoPCs (C18:2, C18:1, C16:0, C17:0, C26:1) were negatively correlated with BMI, WC, and FLI (Figure 6B). Various PCaas correlated positively with triglycerides, blood cholesterol, LDL, HDL, GGT, BMI, and FLI. Negative correlations of PCaas were also observed in blood triglycerides, fasted blood glucose, BMI, WC, and FLI (Figure 6C). No correlation could be found between metabolites and HRI.

## 4. Discussion

In the present study, we examined the metabolic profiles of three different diets which differ in the amount of fructose content (10 vs. 100 g/day) and the derivation of fructose (from complex food sources vs. high fructose syrup). Applying a targeted metabolomics analysis followed by multivariate data analysis has shown that both high fructose diets caused marked effects on various metabolite classes. Furthermore, some differences between the metabolomic shift of lean and obese study subjects were observed in response to the different diets. 

Acylcarnitines play an important role for the transport of acyl-residues into the mitochondrial matrix. As a result of the activity of carnitine-palmitoyltransferase I (CPTI), fatty acids and carnitine bind to acylcarnitines [22]. CPTI is located on the outer mitochondrial membrane and is responsible for the rate-determine step of β-oxidation [23,24]. Acylcarnitine C16:1 was shown to contribute highly to the class separation of different fructose diets. C16:1, also known as hexadecenoyl-l-carnitine, promotes the transport of long-chain fatty acids into mitochondria. A study investigating the effects of two weeks of orange juice consumption concluded that orange juice could affect β-oxidation. A decrease of medium- and long-chain acylcarnitine levels, including C16:1, and an increase of short-chain acylcarnitine levels induced by orange juice was observed [25], which is in accordance with our observations under a high fructose diet. Acylcarnitine C4:1 (butenylcarnitine) was particularly higher during the fruit compared to the low fructose diet. The authors assume that the reaction of fatty acid transportation into the mitochondria and subsequent β-oxidation is incomplete, resulting in an accumulation of chain-shortened acylcarnitines as oxidation intermediates [26]. The high level of butyrylcarnitine (C4), due to incomplete β-oxidation, is assumed to be associated with an increase in reactive oxygen species [27]. In this study, similar results were observed between low fructose and high fructose diet phases. It can be concluded that these findings result from high fructose consumption.

Previous studies suggested an association between acylcarnitines and insulin resistance [28,29]. However, so far, there is no clear evidence that acylcarnitines themselves are involved in the development of insulin resistance [30]. 

High fructose consumption is thought to be involved in the development of experimental liver steatosis [7]. It is assumed that the continuous consumption of soft drinks, often sweetened with “high fructose corn syrup”, is a most relevant risk factor for lipid accumulation into the liver in Non-alcoholic fatty liver disease (NAFLD) patients [31]. In the liver, fructose is metabolized to triglycerides and other metabolites. Additionally, fructose inhibits lipid oxidation in the mitochondria [32]. Both effects promote lipid accumulation in hepatocytes and favor the development of hepatic steatosis [33]. The results of this study, in particular the decreased acylcarnitine concentrations we found, suggest that the β-oxidation might be inhibited by the high fructose diets which is in accordance with the results of other studies reporting a reduced β-oxidation caused by a high fructose load [32,34,35]. This can be explained by a reduced lipolysis and a decreased expression of the transcription factor PPARα, which controls the expression of CPTI as well as further enzymes thought to play an important role in β-oxidation [36,37]. Furthermore, malonyl-CoA, which is synthetized in the hepatocytes during the de novo lipogenesis, has an inhibitory effect on CPTI [33]. Therefore, less activated fatty acids are transported into the mitochondrial matrix. Consequently, less fatty acids can be metabolized via β-oxidation [33].

A decrease in acylcarnitine levels during high fructose diets may reflect a shift in energy production from β-oxidation to glycolysis. This is confirmed by the significant negative correlations of acylcarnitine C18:2, C12:1, and C12:0 with serum triglycerides, which are presumably elevated because of decreased plasma β-oxidation. A reduced fasting β-oxidation represents an unfavorable change in the energy balance of the body, as decreased fatty acid oxidation leads to a positive fatty acid balance and may result in lipid storage in adipocytes and hepatocytes. An increased lipid accumulation in the liver is suggested by the negative correlations we found between several medium- and long-chain acylcarnitines and the liver enzymes GPT and GGT as well as the FLI. This suggestion is further strengthened by the positive correlations we found between short-chain acylcarnitines C3 as well as C5 and the enzymes GPT, GGT and the FLI. Furthermore, the positive correlations between the short-chain acylcarnitines C3 and C5 on the one hand, and anthropometric parameters like BMI and WC on the other, fit to an increased storage of fat into the tissue and thus weight gain.

In response to oxidative stress, phosphatidylcholines of LDL are converted into lysoPCs by the activity of phospholipase A2 (PLA_2_). In addition to lysoPCs, oxidized fatty acids are released [38,39]. In recent years, the importance of lysoPCs for the development of endothelial dysfunction and arteriosclerosis has been studied, as lysoPCs can be found in high concentrations within arteriosclerotic plaques [40,41], with higher concentrations in symptomatic compared to asymptomatic plaques [42]. Furthermore, the development of arteriosclerosis is thought to be based on inflammatory reactions of the vascular cells [43]. LysoPCs play an important role in the initiation and progression of inflammation. Macrophages are triggered for increased IL-1β production by lysoPC resulting in a migration of other immune cells to the affected region and an actuation of the inflammatory response [41]. Additionally, it has been shown that IL-1β induces gene expression and secretion of PLA_2_ that catalyzes the cleavage of modified phospholipids to lysoPCs [44]. On the basis of such data, LysoPCs have been proposed as potential in vivo markers for the progression of atherosclerosis [45].

To our knowledge, biochemical studies on particular lysoPCs metabolites are rare. Therefore, we cannot explain the physiological meaning of the high contribution of lysoPC a C14:0 to the class separation found in our study. 

A study which analyzed the plasma profile of a large population cohort found a strong negative association between lysoPCs and BMI [46]. In the present study, lysoPCs correlated negatively with BMI and WC. It was reported that a reduction in lysoPC level could result from a high fat diet [47]. We did not study the effect of dietary fat in the present study, but our data indicate that fructose can also alter lysoPC levels. Thus, lysoPC levels might be affected by different diet components including fat and fructose. 

Assuming that obese people are exposed to chronic oxidative stress and chronic inflammation [48,49], lysoPCs are expected to be elevated in adiposity [50]. In our study, no significant difference in mean lysoPC level between lean and obese subjects was observable. However, an elevated lysoPC a C16:1 was detected in the high fructose compared to the low fructose group. Various studies reported an increase in oxidative stress in cells induced by fructose [51,52,53]. The appearance of the pro-oxidative state induced by fructose leads to an increased susceptibility of oxidation processes. As a result, oxidized LDL particles are produced and, because of the activity of PLA_2_, plasma lysoPC levels are increased. 

The results obtained in the present study illustrate the antioxidant effect of fruit and vegetable diets. Despite the same total fructose intake during the two high fructose diets (100 g/day), only the consumption of the HFS diet caused a significant increase in plasma lysoPCs in obese subjects, indicating their oxidative influence. In contrast, the fruit diet did not lead to a significant change in plasma lysoPCs. This finding is most likely attributable to the different sources of fructose. Fruits and vegetables are rich in vitamins, minerals, antioxidants, polyphenols, and a good source of dietary fiber in the form of insoluble and soluble fibers. Soluble fibers bind to water and increase the viscosity of food digesta, resulting in a prolonged gastric emptying and delayed absorption of nutrients [54,55,56,57,58], including fructose. Fructose from fruits and vegetables is therefore absorbed and released consistently in smaller amounts, in contrast to fructose from syrup. Another effect of dietary fiber from fruits and vegetables was reported to result in lower blood cholesterol levels [58,59] and plasma lipid concentration [58,60]. Therefore, fiber rich foods improve LDL levels [61] which could lead to a reduced release of lysoPCs from oxidized LDL particles. Additionally, phytonutrients, including vitamins and polyphenols, are mainly located in the fiber of fruits and vegetables. Vitamins and polyphenols are known to have an antioxidant and anti-inflammatory capacity [58]. As mentioned before, fructose was reported to increase oxidative stress in cells [51,52,53]. Antioxidants might prevent the production of oxidized LDL particles and therefore lower lysoPC levels. All these effects may explain the significant increase of lysoPC levels found in the HFS diet but not in the fruit diet. It is also worth mentioning the anti-inflammatory behavior of vitamins and polyphenols, which could lower the pro-inflammatory behavior of lysoPCs.

The present study found a significant difference in the metabolites PCaa C32:1, C34:3, C38:3, C40:4, C40:5, and C40:6 between low fructose and high fructose diets in obese subjects. This strengthens the hypothesis of the importance of PCaas to class separation of the diet phases. Interestingly, the study of Reinehr et al. [62] found a decreased level of PCaa 32:1, C38:3, C40:4, and C40:5 in obese children with a decrease in weight compared to those without weight change. The fact that the same pattern of PCaas are affected by a high fructose diet and by weight change in the obese allows the assumption that these PCaas might serve as biomarkers for diet and weight change in obese individuals. This hypothesis is supported by reports showing that PCaas correlate with various clinical parameters including blood cholesterol levels [63]. Also in our study, some positive correlations between preferentially unsaturated PCaas and plasma parameters like total, LDL, and HDL, triglycerides, and GGT were found. 

A limitation of the study is the small number of subjects. Therefore, we chose the crossover study design which reduces the risk of false positive and negative results because of interindividual variabilities. Moreover, the narrow age range of enrolled women and the strict diet protocol they had to undergo should outbalance confounders such as age, gender, exercise, and diet to some extent. Subjects were asked to report their nutritional intake via a protocol but they were not directly observed for compliance. Therefore, the risk of missing compliance remains. Nevertheless, the metabolomics analysis did show differences between high and low fructose diet phases despite the short-term intervention phases of one week. Therefore, it can be assumed that the compliance of the subjects was not too bad. Since we included only females in the study, we wondered if the estrous cycle has an influence on the metabolite profile. A single recent study investigating metabolome changes during the estrous cycle in women found significant changes in certain metabolite levels in serum, namely free fatty acid C20:4, fatty acyl amide C18:1, tyrosine, and valine [64]. In our study, we did not assess the estrous status of the women, which is a limitation. We cannot rule out the possibility of hormone-dependent influences. Furthermore, it is known that the metabolic profile of men and women differ [63]. Since we only studied fructose effects in females to reduce participant-related variabilities, we cannot draw any scientific conclusions concerning fructose effects on the metabolome in men or in the general population. Another limitation, which is typical for metabolic studies, is the risk of type I errors due to multiple testing. FDR correction was used to reduce this kind of error. 

In conclusion, our study shows that high fructose consumption shifts metabolic patterns towards a constellation that might be associated with an inhibition of mitochondrial β-oxidation and an increase in lipid peroxidation. The effects of fructose on the metabolites tended to be more pronounced following the HFS diet than following the fruit-based diet; however, these differences were not consistent for all metabolites and were more visible in obese rather than lean individuals.

## Figures and Tables

**Table 1:** Baseline characteristics of the study population.

Characteristics	Lean (*n* = 6)	Obese (*n* = 6)	*p*-Value
Age (years)	26 ± 2	30 ± 3	0.013
BMI (kg/m^2^)	22.5 ± 1.5	41.5 ± 4.0	0.004
WC (cm)	72.4 ± 2.5	118.3 ± 9.4	0.004
BP Sys (mmHg)	104.2 ± 7.4	122.5 ± 9.9	0.004
BP Dias (mmHg)	69.2 ± 6.7	83.3 ± 6.8	0.005
FBG (mg/dL)	87.7 ± 4.6	93.3 ± 8.4	ns
HDL (mg/dL)	62.0 ± 11.4	53.8 ± 14.2	ns
LDL (mg/dL)	100.0 ± 22.1	137.2 ± 35.4	0.025
TG (mg/dL)	82.0 ± 20.5	108.7 ± 63.7	ns
FLI	7.0 ± 2.5	89.8 ± 12.5	0.001
HRI	0.9 ± 0.2	1.0 ± 0.1	ns
GGT (U/L)	15.5 ± 2.2	25.5 ± 17.7	ns
GPT (U/L)	16.2 ± 3.1	23.7 ± 6.2	0.024

Data are expressed as mean (± SD). Abbreviations: FBG, fasting blood glucose; BMI, body mass index; BP, blood pressure; FLI, fatty liver index; GGT, gamma-glutamyl-transpeptidase; GPT, glutamate-pyruvate-transaminase; HDL, high-density lipoproteins; HRI, hepatorenal index; LDL, low-density lipoprotein; TG, triglycerides; WC, waist circumference.

**Table 2:** Composition of study diets.

Nutrients	Low Fructose Diet		Fruits		HFS	
	Goal	Actual Intake	Goal	Actual Intake	Goal	Actual Intake
**Energy (kcal/day)**						
Lean	2002 ± 56	1828 ± 260	2005 ± 54	2188 ± 357	2006 ± 59	2404 ± 404 *
Obese	2207 ± 51	1949 ± 442	2209 ± 51	2404 ± 410	2210 ± 51	2961 ± 273 *
**Protein (g/day)**						
Lean	73 ± 2	83 ± 10	73 ± 2	69 ± 15	73 ± 2	65 ± 10
Obese	81 ± 2	102 ± 41 *	81 ± 2	91 ± 29	81 ± 2	92 ± 28
**Fat (g/day)**						
Lean	65 ± 2	80 ± 10 *	65 ± 2	67 ± 20	65 ± 2	70 ± 20
Obese	71 ± 2	89 ± 25 *	71 ± 2	79 ± 24	71 ± 2	93 ± 18
**CHO (g/day)**						
Lean	268 ± 8	176 ± 52 *	271 ± 5	308 ± 45	271 ± 5	357 ± 58
Obese	296 ± 7	174 ± 24 *	288 ± 4	314 ± 24	288 ± 4	416 ± 47
**Fiber (g/day)**						
Lean	medium	17 ± 6	medium	36 ± 8	low	12 ± 5
Obese	medium	19 ± 8	medium	39 ± 7	low	19 ± 7
**Complex CHO (g/day)**						
Lean	258 ± 8	160 ± 48 *	171 ± 5	128 ± 5 *	171 ± 5	121 ± 30 *
Obese	286 ± 7	166 ± 19 *	188 ± 4	131 ± 3 *	188 ± 4	174 ± 32
**Sac (g/day)**						
Lean	low	8 ± 2	medium	55 ± 13	low	10 ± 7
Obese	low	4 ± 3	medium	59 ± 14	low	10 ± 7
**Fru (g/day)**						
Lean	10	7 ± 2	100	100 ± 12	100	106 ± 11
Obese	10	4 ± 3	100	102 ± 4	100	108 ± 6
**Glu (g/day)**						
Lean	low	7 ± 2	medium	80 ± 8	high	130 ± 12
Obese	low	5 ± 3	medium	80 ± 9	high	134 ± 6

Data are expressed as mean (± SD). * More than 20% difference between the proposed amount and the actual intake. Abbreviations: CHO, carbohydrates; Fru, fructose; Glu, glucose; HFS, high fructose syrup; Sac, saccharose.

## Supplementary Materials

The following are available online at http://www.mdpi.com/2072-6643/10/9/1254/s1, Table S1: Metabolite concentrations of low and high fructose diets.
