# Supplementary material for: Changes in Plasma Acylcarnitine and Lysophosphatidylcholine Levels Following a High-Fructose Diet: A Targeted Metabolomics Study in Healthy Women
Source: Nutrients. 2018 Sep 6;10(9):1254. doi: 10.3390/nu10091254 (PMC6165514; doi:10.3390/nu10091254)
Supplement: Supplementary file 1 [file nutrients-10-01254-s001.pdf]

Table S1. Metabolite concentrations of low and high fructose diets.

Metabolite class	Metabolite [µmol/L]	Low fructose (n = 12)	High fructose (n = 12)	Adj. p -Value
diacyl-phosphatidylcholine	PC aa C32:1	15.46 ± 7.91	24.86 ± 10.24	< 0.001
diacyl-phosphatidylcholine	PC aa C38:3	41.92 ± 8.05	51.97 ± 9.24	< 0.01
acylcarnitine	C2	5.97 ± 1.6	4.43 ± 1.0	< 0.01
lysophosphatidylcholine	lysoPC a C14:0	5.11 ± 0.55	5.86 ± 0.59	< 0.01
acylcarnitine	C18:2	0.03 ± 0.01	0.02 ± 0.008	< 0.01
diacyl-phosphatidylcholine	PC aa C34:1	210.58 ± 47.35	255.17 ± 52.31	< 0.01
acylcarnitine	C16:1	0.04 ± 0.01	0.03 ± 0.003	< 0.01
diacyl-phosphatidylcholine	PC aa C40:5.t	8.34 ± 1.88	10.89 ± 2.48	< 0.01
acylcarnitine	C18:1	0.1 ± 0.02	0.08 ± 0.02	< 0.01
acylcarnitine	C7-DC	0.04 ± 0.01	0.03 ± 0.01	< 0.01
diacyl-phosphatidylcholine	PC aa C30:0	4.62 ± 1.21	6.44± 1.89	< 0.01
lysophosphatidylcholine	lysoPC a C16:1	2.29 ± 0.67	2.99 ± 0.79	< 0.01
acylcarnitine	C10	0.34 ± 0.13	0.25 ± 0.06	< 0.01
acylcarnitine	C14:2	0.03 ± 0.02	0.02 ± 0.01	< 0.01
acylcarnitine	C12	0.12 ± 0.04	0.09 ± 0.02	< 0.01
acylcarnitine	C14:1-OH	0.01 ± 0.003	0.01 ± 0.002	< 0.01
diacyl-phosphatidylcholine	PC aa C40:6	22.8 ± 7.36	26.77 ± 7.68	< 0.01
acylcarnitine	C16:2	0.01 ± 0.002	0.004 ± 0.001	< 0.01
acylcarnitine	C14:1	0.13 ± 0.03	0.11 ± 0.02	< 0.01
diacyl-phosphatidylcholine	PC aa C36:3	125.91 ± 33.69	141.65 ± 34.49	< 0.01
diacyl-phosphatidylcholine	PC aa C34:3	15.2 ± 6.71	17.86 ± 6.49	< 0.01
acylcarnitine	C8	0.2 ± 0.06	0.17 ± 0.03	< 0.05
acylcarnitine	C12:1	0.19 ± 0.04	0.16 ± 0.03	< 0.05
diacyl-phosphatidylcholine	PC aa C40:4	3.71 ± 0.97	4.74 ± 1.36	< 0.05
amino acid	serine	94.36 ± 18.81	81.77 ± 13.92	< 0.05
acylcarnitine	C14	0.05 ± 0.01	0.04 ± 0.006	< 0.05
acyl-phosphatidylcholine	PC ae C30:0	0.44 ± 0.12	0.52 ± 0.14	< 0.05
acylcarnitine	C10:1	0.18 ± 0.04	0.2 ± 0.06	< 0.05
acylcarnitine	C6 (C4:1-DC)	0.06 ± 0.02	0.05 ± 0.008	< 0.05
diacyl-phosphatidylcholine	PC aa C32:2	4.04 ± 1.71	5.07 ± 1.22	< 0.05
diacyl-phosphatidylcholine	PC aa C34:4	1.98 ± 0.86	2.49 ± 0.61	< 0.05
diacyl-phosphatidylcholine	PC aa C36:1	51.78 ± 12.27	64.62 ± 17.16	< 0.05
acylcarnitine	C16	0.09 ± 0.02	0.08 ± 0.01	< 0.05
acyl-phosphatidylcholine	PC ae C34:1	10.71 ± 2.0	12.22 ± 1.98	< 0.05
lysophosphatidylcholine	lysoPC a C16:0	65.01 ± 13.89	76.13 ± 17.36	< 0.05
lysophosphatidylcholine	lysoPC a C20:3	1.73 ± 0.49	2.05 ± 0.46	< 0.05
acylcarnitine	C4:1	0.021 ± 0.002	0.019 ± 0.002	< 0.05
acylcarnitine	C5	0.1 ± 0.04	0.09 ± 0.03	< 0.05

Data are expressed as mean (± SD).
